# Surveillance cues do not enhance altruistic behavior among anonymous strangers in the field

**DOI:** 10.1371/journal.pone.0197959

**Published:** 2018-08-16

**Authors:** Erik J. Koornneef, Aurelie Dariel, Iffat Elbarazi, Ahmed R. Alsuwaidi, Paul B. M. Robben, Nikos Nikiforakis

**Affiliations:** 1 Institute for Health Policy and Management, Erasmus University, Rotterdam, The Netherlands; 2 Division of Social Science, New York University Abu Dhabi, Abu Dhabi, UAE; 3 Institute of Public Health, UAE University, Al Ain, UAE; 4 Department of Pediatrics, UAE University, Al Ain, UAE; Universidad Loyola Andalucia, SPAIN

## Abstract

The degree of altruistic behavior among strangers is an evolutionary puzzle. A prominent explanation is the evolutionary legacy hypothesis according to which an evolved reciprocity-based psychology affects behavior even when reciprocity is impossible, i.e., altruistic behavior in such instances is maladaptive. Empirical support for this explanation comes from laboratory experiments showing that surveillance cues, e.g., photographs of watching eyes, increase altruistic behavior. A competing interpretation for this evidence, however, is that the cues signal the experimenter’s expectations and participants, aware of being monitored, intentionally behave more altruistically to boost their reputation. Here we report the first results from a field experiment on the topic in which participants are unaware they are being monitored and reciprocity is precluded. The experiment investigates the impact of surveillance cues on a textbook example of altruistic behavior—hand hygiene *prior* to treating a ‘patient’. We find no evidence surveillance cues affect hand hygiene, despite using different measures of hand-hygiene quality and cues that have been previously shown to be effective. We argue that surveillance cues may have an effect only when participants have reasons to believe they are actually monitored. Thus they cannot support claims altruistic behavior between strangers is maladaptive.

## Introduction

The degree of altruistic behavior among strangers in modern societies is a major evolutionary puzzle [1,2]. A prominent explanation is the ‘evolutionary legacy hypothesis.’ It posits that the human brain evolved in ancestral conditions that differed radically from those in modern environments [3,4]. Although nowadays many encounters are with anonymous strangers, for much of our evolutionary past, humans interacted repeatedly in small social groups where one’s reputation was constantly at stake, leading to the evolution of cognitive mechanisms to automatically identify reputation-building opportunities [5–7]. According to the evolutionary legacy hypothesis, individuals may behave altruistically in anonymous one-shot interactions due to an uncontrolled, automatic reaction aimed to bolster one’s good reputation in anticipation of positive reciprocity, even when such opportunities do not exist; i.e., the observed altruistic behavior between anonymous strangers is maladaptative [8,9].

Empirical support for this hypothesis comes from laboratory experiments showing that reputation-related surveillance cues such as displaying photographs of watching eyes promote altruistic behavior, i.e., actions which benefit another individual at a personal cost [10–12]. Since the cues do not affect one’s reputation, participants are anonymous to each other and direct reciprocity is precluded by design, the effect has been attributed to the automatic activation of one’s reciprocity-based psychology [8,9]. A problem with this interpretation, however, is that participants are not anonymous to the experimenter who may be observing their choices or can easily infer them from their earnings. A competing interpretation therefore is that the increase in altruism is due to an ‘experimenter effect’ [13–15]: surveillance cues signal the experimenter’s expectations to participants who intentionally react to the stimulus in a way they believe would boost their reputation with him/her. In other words, even though direct reciprocity between participants is precluded, indirect reciprocity concerns may still play a role. In line with this interpretation, survey evidence shows the effect on altruistic behavior is mediated by participants’ expectation of reward by “a third party who was monitoring them” [16].

One way to eliminate the possibility of an experimenter effect is to conduct field experiments such that individuals are unaware they are participating in a study. In order to provide clear evidence that altruistic behavior is maladaptive, however, certain conditions need to be satisfied such that alternative explanations are ruled out. In particular, it is critical that all encounters in the experiment are anonymous, one-shot and reciprocity of any kind is precluded. It is also desirable that exposure to the cues is brief as habituation with the false stimulus may attenuate the effect through intentional brain processes [7,12]. If surveillance cues are found to increase altruistic behavior in such circumstances this would support the hypothesis that altruistic behavior between anonymous strangers is maladaptive. If surveillance cues have no effect, it would suggest that previous findings may have been due to an *intentional* decision taken to bolster one’s reputation with the experimenter. Here, we present the first evidence from such a field experiment.

We take advantage of a unique opportunity to study altruistic behavior in a setup which meets all the aforementioned requirements. This distinguishes our experiment from previous field studies in which surveillance cues were displayed in *public* spaces over an *extended* period of time [17–25]. As we explain in the last section of our paper, these studies were designed to address different research questions. As such, the positive effect of surveillance cues on altruistic behavior in these studies suggests a potentially useful, low-cost, policy intervention, but it cannot support the claim that altruistic behavior among strangers is maladaptive as many of the aforementioned conditions are not satisfied and indirect reciprocity opportunities exist.

In our experiment we investigate how two distinct surveillance cues impact the quality of hand hygiene by medical students before treating a ‘patient.’ Hand hygiene (HH) is a general term used to describe the process of removing microorganisms with a disinfectant agent such as alcohol, or soap and water [26]. Appropriate HH among healthcare workers is considered by some to be the most effective measure to prevent healthcare-associated infections [27], which are associated with 50,000 and 99,000 deaths each year in Europe and the USA, respectively [28], and annual hospital costs between $28.4 to $33.8 billion USD [26]. It is estimated that a one-percent improvement in the quality of hand hygiene could save approximately $40,000 USD per year in a 200-bed hospital for a single type of infection [29]. That is, *how* one washes his or her hands is critical. Accordingly, compliance with HH guidelines has been identified by the World Health Organization (WHO) as a first priority in health-care facilities [28].

Appropriate HH *prior* to treating a patient is a textbook example of altruistic behavior. According to WHO’s guidelines, when contact with a patient is not invasive—as is the case in our experiment—a healthcare provider must follow a specific technique to thoroughly wash his/her hands both before and after contact, for 40 to 60 seconds each time (when contact is invasive, the duration should be between 120 to 300 seconds) [28]. HH prior to treating a patient is not only costly, taking time and effort, but also it does not benefit the healthcare provider directly, only the patient whose chances of a healthcare associated infection are estimated to decrease between 15 and 30% [30]. Both the cost to the practitioner [31] and the lack of individual benefits [32] from HH prior to treating a patient have been cited as prime reasons for why compliance with WHO’s guidelines is low. In support of the idea that HH prior to treating a patient constitutes an altruistic act is the evidence that compliance with HH guidelines is substantially higher *after* contract with the patient [31,33]. In the concluding section, we present results from a survey showing that concerns for the welfare of the patient indeed appear to be the primary reason for washing hands prior to treating a patient in our experiment.

## The experiment

The experiment was conducted in a large university, which is well-regarded locally for its medical program: the United Arab Emirates University (see section A in S1 Appendix). Participants were advanced undergraduate students in the Doctor of Medicine (MD) program who had completed training modules in the basic principles of clinical practice, including infection prevention and HH in accordance with WHO guidelines. For the experiment, we took advantage of a unique opportunity offered by the program for students to *privately* practice their clinical skills—a *Practice* Objective Structured Clinical Examination (POSCE). The *official* OSCE is a critical part of all MD programs aimed to formally evaluate one’s clinical competence. Medical students in the OSCE are observed and evaluated by faculty members as they go through a series of stations, interviewing, examining and treating different standardized patients who present some type of medical problem. The POSCE was identical to the OSCE, with two crucial differences: (*i*) faculty members were not present to observe or evaluate the competence of the students, and (*ii*) participants remained anonymous throughout the process. Students were fully aware that the purpose of the exercise was for them to practice their skills without being judged or evaluated.

A note outside the ‘patient’s room’ informed students that their main task was to take the blood pressure of a standardized patient (see section D in S1 Appendix). Medical students are aware that, whenever they are having physical contact with a standardized patient, there is a real risk of contaminating him/her. Participants therefore know that best clinical practice requires they wash their hands carefully immediately prior to measuring the standardized patient’s blood pressure, following the WHO’s HH guidelines. At the same time, participants are not monitored and, like with the OSCE, each practice slot lasts ten minutes—this is signified by automated bells in the corridors, which were meant to reinforce the fact that the POSCE was not monitored. During this time, they had to briefly interview the standardized patient, wash their hands, measure blood pressure, wash their hands again, and provide feedback to the patient. Given their limited experience with the blood measurement instruments, participants had an incentive to take advantage of this one-off opportunity and spend most of their time practicing measuring blood pressure as it is likely to be relevant in the official OSCE. Therefore, there is a non-trivial cost for participants from properly washing their hands, but incurring the cost benefits the standardized patient. It should be noted that students could not benefit patients by expediting HH as each POSCE slot lasted *exactly* ten minutes, i.e., they could leave neither earlier nor later.

**Fig 1:**
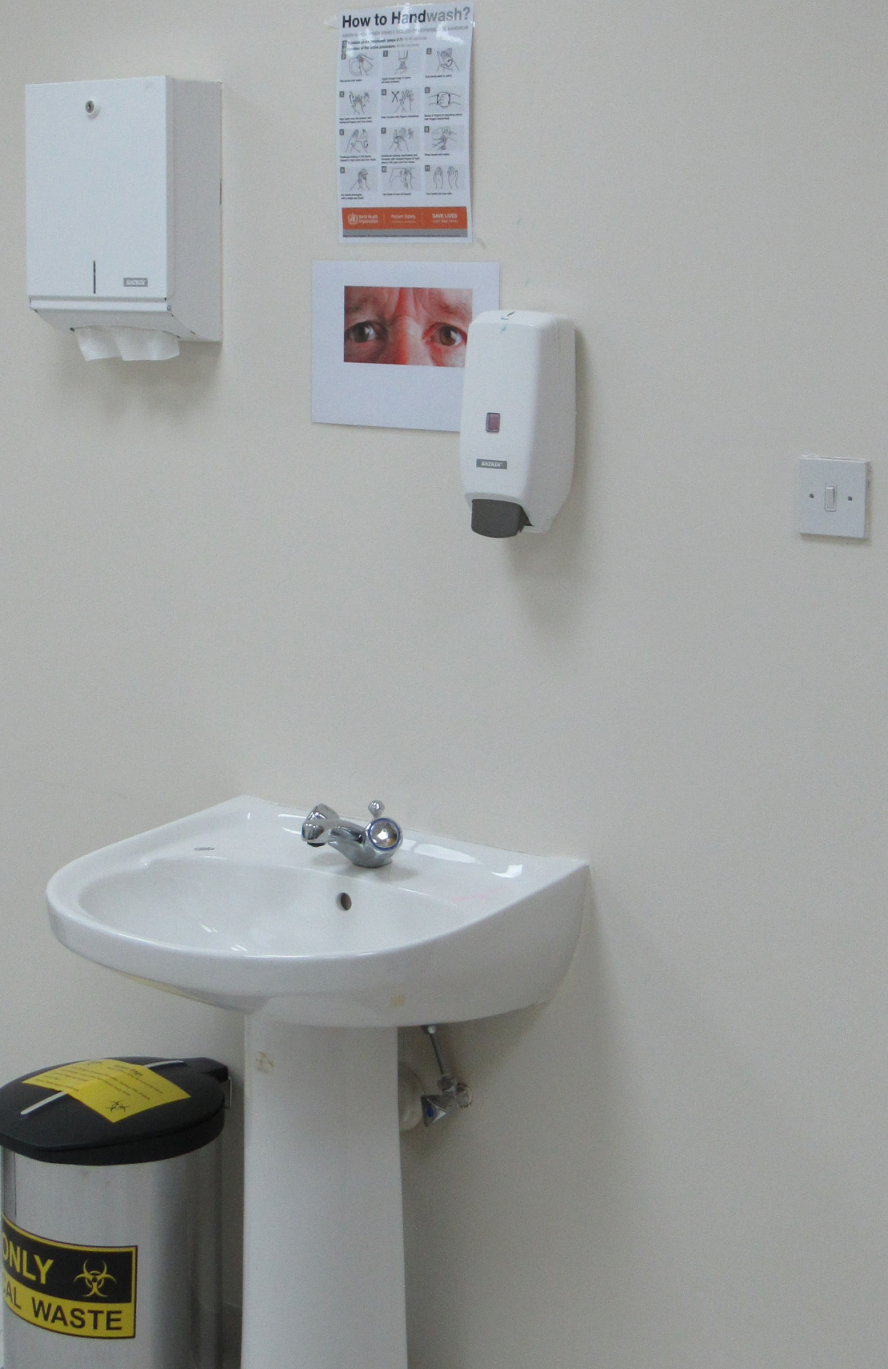
Picture of the wash basin in the private examination room featuring the watching eyes.

The experimental treatments varied the surveillance cues which were displayed, approximately at eye-level, above a wash basin (see Fig 1, and section G in S1 Appendix) and underneath the standard HH poster by WHO explaining in detail appropriate HH (see section H in S1 Appendix). Due to the HH guidelines and the limited time of the session, exposure to the surveillance cue was necessarily brief (<60 seconds). Participants were randomly assigned to treatments/cues (see section A in S1 Appendix). The *Baseline* condition, like previous studies, consists of a non-reputation-related image—the picture of a tree. In the *Eyes* treatment, a pair of stern-looking male eyes was displayed. This particular image was chosen as it has been previously associated with a large positive effect on HH, i.e., a 122% increase relative to a baseline condition when the cue was placed in a public space over an extended time period [25]. This was important as it was uncertain ex ante how large a sample we could hope to attract. Ultimately, the turnout was substantial and higher than we had expected: 114 students out of an eligible student population of 330. With this sample size, our tests have sufficient power to detect treatment differences substantially smaller than those in King et al. [25] (see section C in S1 Appendix). Note that in order to be exposed to the treatment manipulation, participants had to go to the wash basin to wash their hands. Some participants in our sample had to be prompted to do so by the standardized patient. Our results are unaffected if we exclude these participants from the analysis (see section B in S1 Appendix).

The *Camera* treatment is the first of its kind in the literature. In this treatment, the picture of a CCTV camera was placed over the wash basin. Such CCTV cameras are omnipresent in the country of our study, although none was available in the examination room. If people have developed cognitive mechanisms to *automatically* identify reputation-building opportunities through millennia, and this is the cause for the changes in altruistic behavior when reputation-related cues are presented, then we would expect to observe an effect in the Eyes but not in the Camera treatment [8]. A difference in HH between our Camera and Baseline conditions could be interpreted as evidence participants are concerned about actually being watched.

Apart from informing students they would need to measure blood pressure, the briefing note outside the room explained that the only people inside the room will be two simulated patients that will take turns being the ‘patient’. (Rooms were spacious: approx. 25 square meters or 270 square feet, see section F in S1 Appendix.) This is standard practice as a person should not have his/her blood pressure measured repeatedly. The simulated patients (RAs) were trained to appear indifferent to the actions of the medical students. At any given point, one RA waited for his/her blood pressure to be measured, and the other–seated at a faraway corner of the room—waited for his turn, while filling out a Sudoku book. In actuality, this individual was covertly monitoring the student’s HH practice (see sections E in S1 Appendix). All RAs had been professionally trained on how to evaluate the quality of one’s HH. Crucially, the simulated patients were selected such that they were completely unknown to the students (with one exception, see section B in S1 Appendix).

Like in previous studies, direct reciprocity is prevented by design: not only were standardized patients in a passive role, but they were also trained to appear bored and indifferent to the POSCE. To preclude indirect reciprocity, encounters had to be anonymous such that reputational concerns could not affect altruistic behavior. For this reason, students and simulated patients were explicitly instructed not to share their identities. As both ‘patients’ would remain in the room at the end of the session to receive the next medical student, while the practicing student would leave, it was clear to participants that encounters were one-shot and that there would be no opportunities for the ‘patients’ to reciprocate, either directly or indirectly. Further, to ensure observers were blind to our treatment manipulation, the RA who acted as the patient changed the poster before the next participant entered the room so that the observer was not aware which poster was displayed at any point in time.

## Results

Our measure of altruistic behavior is the quality of hand hygiene prior to treating the patient. As mentioned, how a medical practitioner washes his/her hands is of critical importance for minimizing the risk of an infection. The survey evidence presented in the concluding section suggests that participants were well aware of this. In order to evaluate the quality of hand hygiene amongst participants, we use three distinct measures from the WHO guidelines about HH. First, we consider the time spent washing hands. Second, we study the quality of hand coverage, i.e., the extent to which a participant washed all surfaces of his/her hands. Third, we consider compliance with a rule prescribing participants use a tissue to switch off the tap, after finishing washing their hands. For simplicity and brevity, we present the means of these variables in the figures below; the distributions of these variables can be found in section K in S1 Appendix.

**Fig 2:**
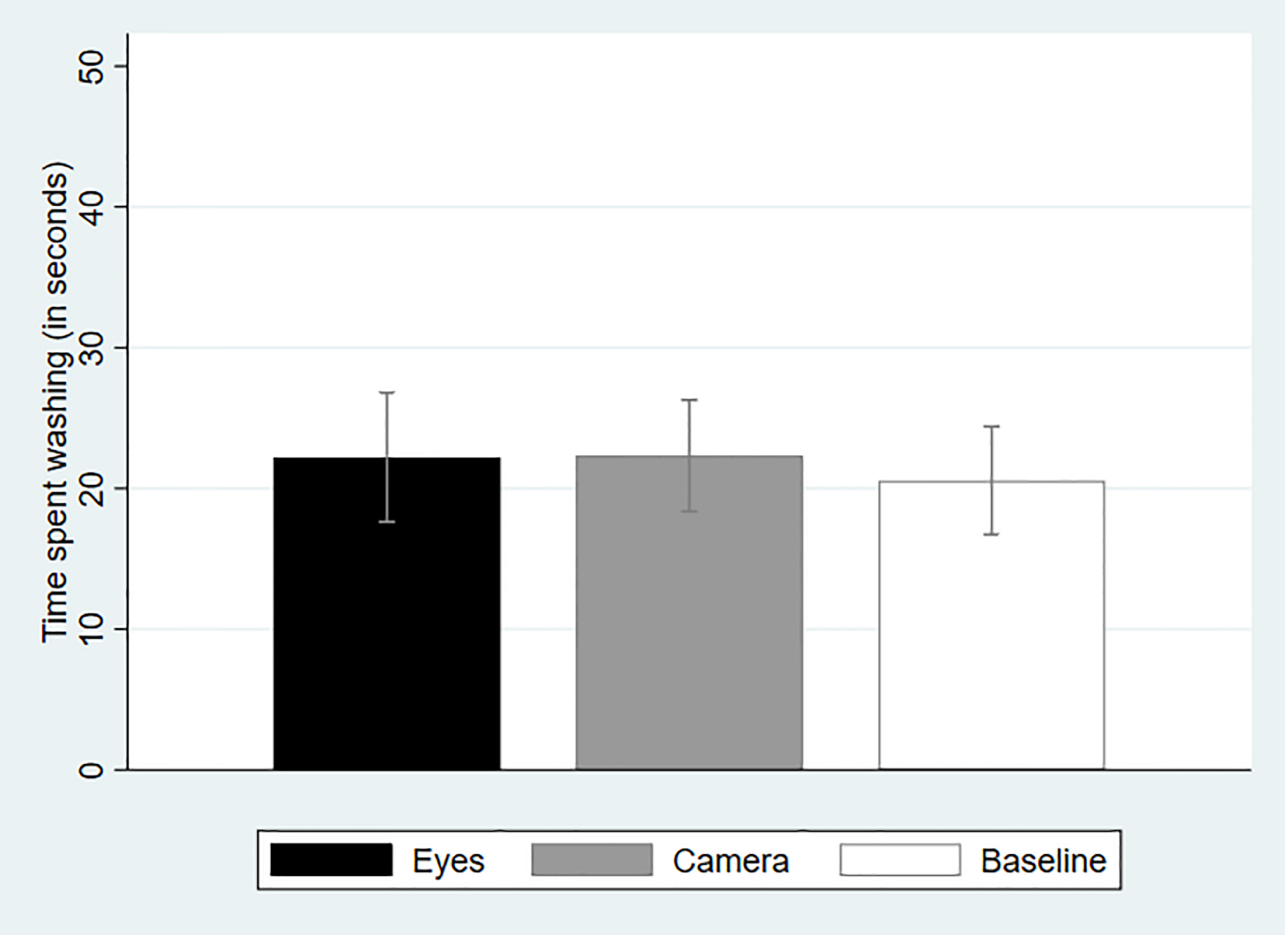
Average time spent washing hands across treatments (with 95-percent confidence intervals).

Fig 2 shows that, across treatments, participants on average washed their hands for slightly more than 20 seconds. While this may be more than the time spent by many adults washing their hands, it falls considerably short of the minimum recommended duration stated by WHO (40 seconds). This implies that there are good conditions for our treatments to increase the quality of hand hygiene and, in this instance in particular, the time spent washing. However, we find no statistically significant differences across treatments (Eyes vs. Baseline: *P* = 0.69, *N* = 71; Camera vs. Baseline: *P* = 0.64, *N* = 79; Mann-Whitney Test, two-tailed).

**Fig 3:**
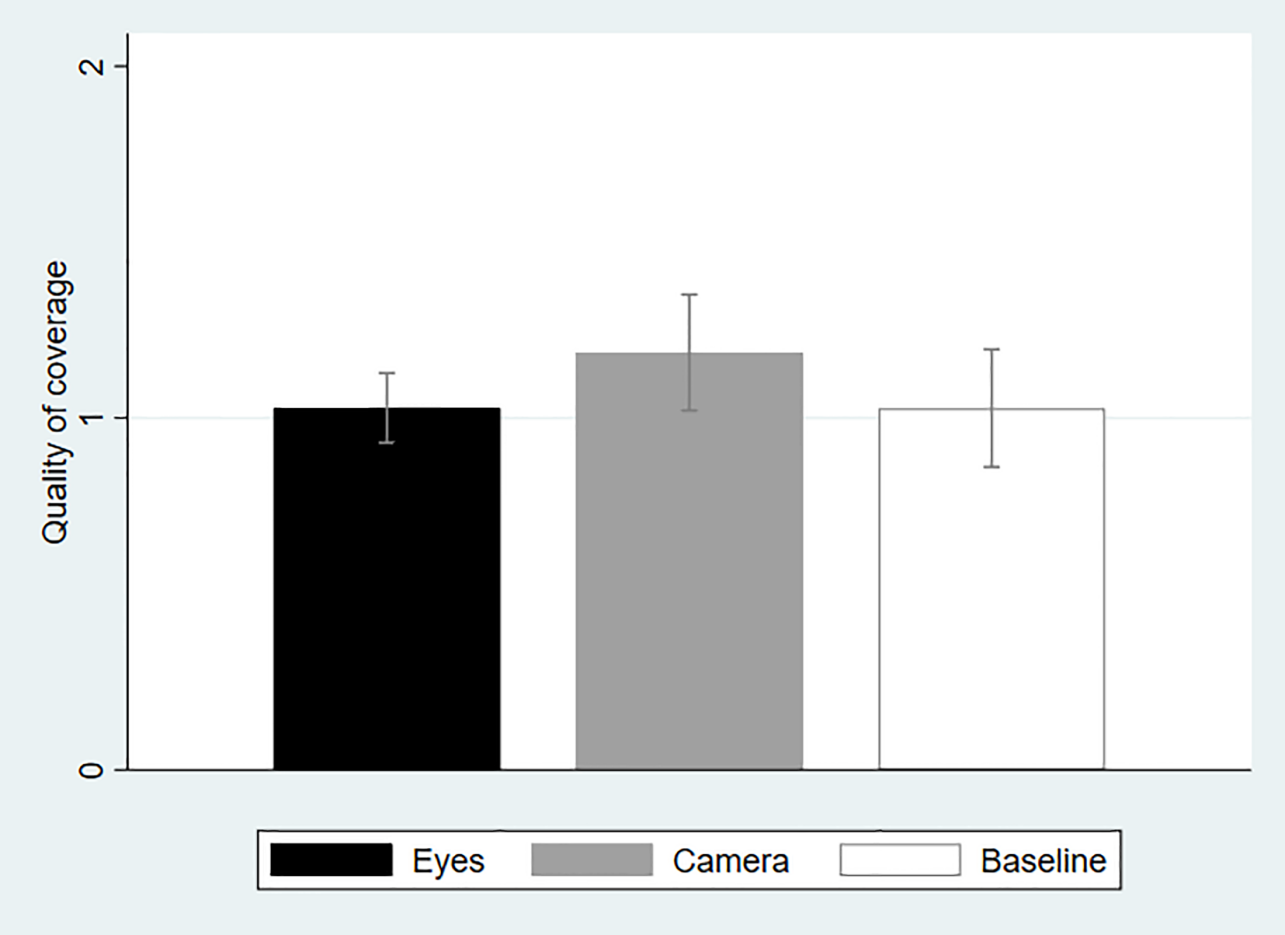
Average quality of hand coverage across treatments (with 95-percent confidence intervals).

Fig 3 presents the average quality of hand coverage across treatments. As mentioned, the RAs were professionally trained to evaluate the extent to which participants followed the WHO guidelines and, in this instance, covered adequately all hand surfaces. Performance was coded as 0 if the participant did not attempt to cover multiple surfaces (e.g., did a simple rub of the palms against each other), as 1 if the participant covered multiple but not all surfaces (e.g., did not wash thumbs), and 2 if the participant covered all surfaces. As can be seen in Fig 3, average coverage is very similar in Eyes and Baseline, and statistically indistinguishable (*P* = 0.99, *N* = 71; Mann-Whitney Test, two-tailed). Although the quality of coverage is slightly higher in the Camera treatment, the difference with Baseline is statistically insignificant (*P* = 0.18, *N* = 79; Mann-Whitney Test, two-tailed).

**Fig 4:**
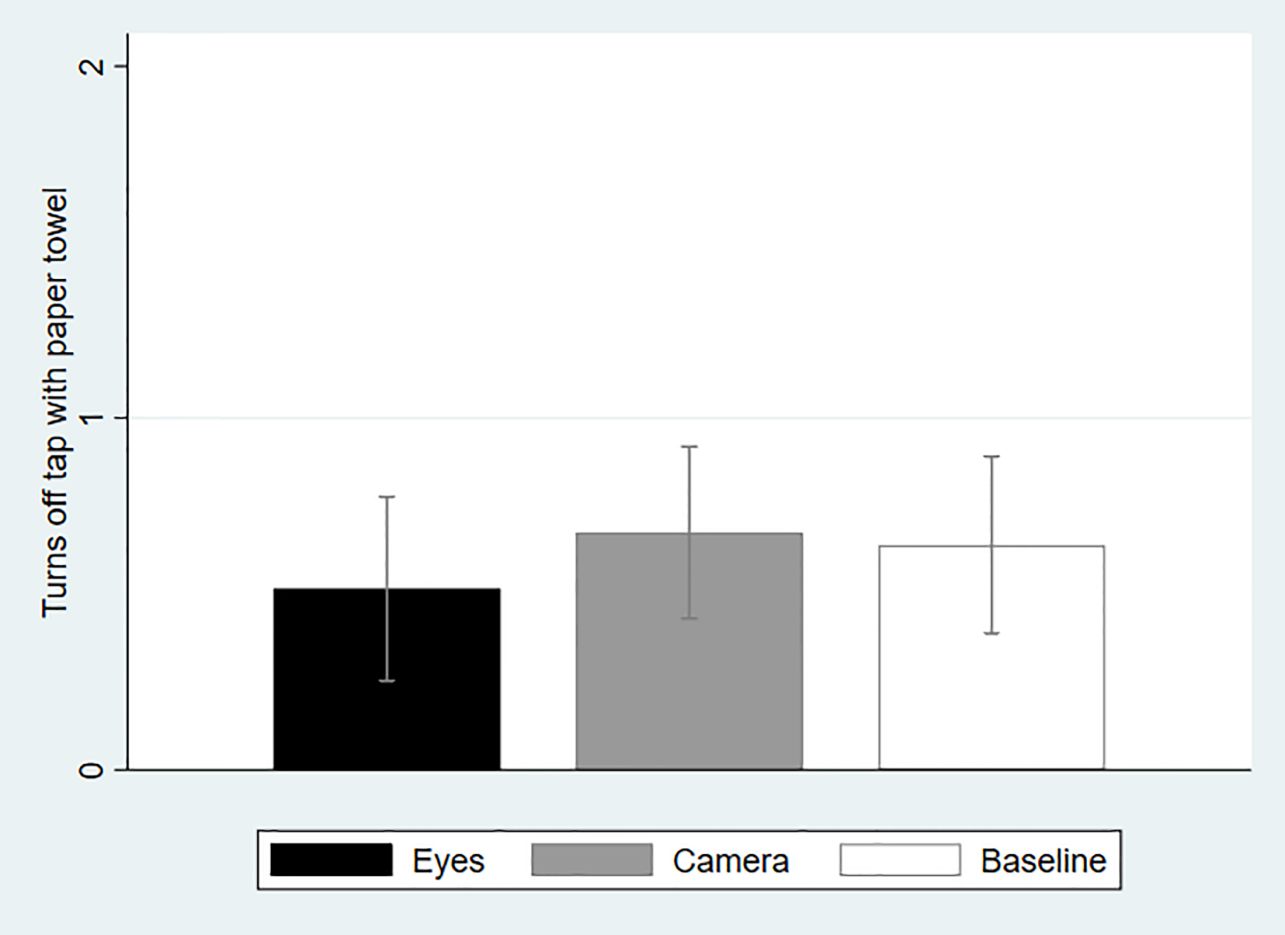
Average compliance with turning-off-tap-with-paper-towel rule across treatments (with 95-percent confidence intervals).

Fig 4 presents the extent to which participants across treatments turned off the tap after washing their hands using a paper towel. This is critical in HH because a lot of bacteria can be found on the water tap. Participants have therefore been trained that not using a towel reduces considerably the efficacy of HH at combating disease transmission. Performance was coded as 0 if the participant did not use a paper towel at all, as 1 if the participant used a paper towel but improperly (e.g., used paper towel but also touched tap with bare hands), and 2 if the participant used properly a paper towel. Average compliance with this rule is lower in Eyes than in Baseline, although the difference is again statistically insignificant (*P* = 0.39, *N* = 71; Mann-Whitney Test, two-tailed). Compliance is similar in Camera and Baseline and statistically insignificant (*P* = 0.90, *N* = 79; Mann-Whitney Test, two-tailed). Neither the fraction of participants using a paper towel properly differs significantly across treatments (Eyes: 17.1%, Camera: 20.9%, Baseline: 16.7%; Eyes vs. Baseline: *P* = 1.00, *N* = 71; Camera vs. Baseline: *P =* 0.78, *N* = 79; Fisher Exact Test, two-tailed) nor the fraction of participants not using a towel at all (Eyes: 65.7%, Camera: 53.5%, Baseline: 52.7%; Eyes vs. Baseline: *P* = 0.37, *N* = 71; Camera vs. Baseline: *P =* 1.00, *N* = 79; Fisher Exact Test, two-tailed).

## Discussion

Our paper presents the first empirical test of the impact of surveillance cues on the altruistic behavior of anonymous strangers when reciprocity is precluded and participants are unaware they are being studied. These conditions are critical to obtain clear support for the evolutionary legacy hypothesis—a prominent explanation for altruistic behavior between strangers—according to which costly altruistic behavior in anonymous encounters is an anomaly, owning to our ancestral past and the development of automated, involuntary mechanisms for boosting one’s good reputation. Despite using cues that have been successfully used previously in the literature, we find no evidence surveillance cues increase the degree of altruistic behavior in our experiment. That is, our findings do not support the hypothesis that altruistic behavior among strangers is maladaptive.

One concern with all studies reporting null results is that this is due to the statistical tests being underpowered. This is clearly not the case in our experiment. Not only do we find no evidence across three distinct measures that the picture of eyes has a significant impact on altruistic behavior in our experiment, but the effect itself is sometimes zero (Fig 3) or negative (Fig 4). By comparison, the effect of posting a picture of a camera over a wash basin—which as we argued could not be considered as supportive of the evolutionary legacy hypothesis—is also always insignificant and small in size, but at least it is always positive. Therefore, the overall lack of a significant effect cannot be attributed to insufficient statistical power.

Another concern may be that the lack of a positive effect is due to the fact that our experiment investigates the impact of surveillance cues on the quality of hand hygiene (intensive margin) but not on the decision to wash hands (extensive margin). Indeed, early evidence from dictator game experiments—in which an individual is assigned an amount of money and must decide how much of it to share with a passive recipient—suggested that surveillance cues may have a greater impact on the likelihood a ‘dictator’ shares a positive amount (extensive margin) than on the actual amount they share (intensive margin); combining margins the effect was often zero [11]. A recent meta-analysis of laboratory studies however contradicts these earlier results, finding no differential effect of cues on the extensive and the intensive margin [34]. Further, some field studies find the opposite result, i.e., that the impact of the cues is stronger on the intensive margin [19] or that a positive effect on charitable donations is obtained even when there are no differences in the proportion of donors responding to the cues [13]. Therefore, there exist neither clear empirical evidence nor theoretical reasons to expect the automatic activation of the reciprocity-based psychology will operate differently on the decisions on the extensive and intensive margins.

Although our design precludes both direct and indirect reciprocity by ensuring encounters are one-shot and all individuals involved (both participants and standardized patients) are unknown and anonymous to each other, we cannot rule out the possibility that, despite our efforts to avoid this, the presence of the standardized patients may have activated participants’ reciprocity-based psychology already in the Baseline treatment, making it difficult to identify a treatment effect. However, it is worth emphasizing that similar concerns apply in laboratory environments. In fact, they are arguably greater: not only there are several participants in the lab *at the same time–*some of whom subjects may know personally—but their decisions are recorded by a computer and possibly observed by the experimenter. Even if this is not the case, participants—who often partake repeatedly in lab experiments—should anticipate that their final payment will ultimately reveal the extent of their altruistic behavior to the experimenter. If the reason for not observing a surveillance-cue effect in our experiment is the activation of the reciprocity-based psychology already in the Baseline condition, then it follows that the lab evidence on “watching eyes” cannot provide clear support either that altruistic behavior among strangers is maladaptive. It should also be noted that behavior across measures in our experiment falls considerably short of that described in the WHO guidelines. If participants were concerned about their reputation, one might have expected higher compliance with the guidelines than observed.

An altogether different concern with our study could be that hand hygiene prior to treating a patient is in fact *not* an altruistic act as we claimed. Although similar claims are common in the medical literature [31,33], one might wonder whether hand hygiene is regarded as altruistic, i.e., as conferring a benefit to the patient, in our particular context by medical students such those participating in our experiment. To address this concern, we administered a survey to 100 medical students with the same level of training and background as those who participated in our experiment (see section I in S1 Appendix). Respondents were presented with a vignette designed to mimic the situation and incentives in our experiment, and asked whether they would wash their hands prior to treating the patient or not, and the reasons for their decisions. The survey also included a question to evaluate our claim that the quality of hand-hygiene matters when it comes to reducing infection by asking participants whether they agree that washing hands for longer reduces the risk of infection for the patient.

Of the survey respondents who stated they would wash their hands prior to taking the blood pressure, 96.6% agreed with the statement that they would do so to avoid doing harm to the patient. We can reject the hypothesis that respondents neither agree nor disagree with the statement in favor of the alternative hypothesis that they agree with it (*P*<0.01, *N* = 87, Wilcoxon signed rank, two tailed). This supports our interpretation of hand hygiene prior to treating a patient as being an altruistic act as it is driven by a concern for the welfare of the patient. Participants were also more likely to agree with this reason for hand washing than with any of the other reasons (*P*<0.01 for all pairwise comparisons, *N* = 87, Wilcoxon signed rank, two tailed) indicating that the desire to do no harm to the patient is the main concern driving hand hygiene prior to contact with the patient. Perhaps unsurprisingly, we find that other concerns also appear to play a role (see sections I and J in S1 Appendix), implying that hand hygiene prior to treatment is not driven exclusively by altruistic concerns for everyone. This, however, does not invalidate our analysis which only requires that altruistic motives are an important determinant of behavior in our experiment. We can also reject the hypothesis that respondents neither agree nor disagree with this statement in favor of the alternative hypothesis that they agree with it (*P*<0.01, *N* = 93, Wilcoxon signed rank, two tailed).

At first pass, our findings appear to contradict those in previous field studies finding a strong positive effect of surveillance cues on altruistic behavior in natural environments [17–25]. Such interpretation of our findings however would be wrong. A critical difference between these studies and ours, stemming from the different research aims, is that the cues in these studies were placed in public spaces such as university cafeterias [17,18], public car parks [20], super markets [13,21] or hospital entrances [25], over an extended period of time. This implies that real reputational concerns were at play. For example, participants in all these studies could self-select into several treatments, more than once, suggesting that individuals may be aware of the treatment manipulations and thus suspect they are being monitored. Similarly, since the manipulations occurred in places frequented by the participants, many of the encounters were likely to be neither anonymous nor one-shot, implying that reciprocity is not precluded by design. For these reasons, these studies suggest a potentially useful, low-cost, policy intervention (as was intended by the authors) but the evidence cannot inform the debate of whether altruistic behavior between strangers is maladaptive.

Taken together, the field evidence suggests that surveillance cues may be effective in promoting altruistic behavior in circumstances in which there are *real* opportunities to build a good reputation. In these instances, the cues may serve as a signal of what the expected behavior is and that behavior is monitored. In line with this is the finding that the surveillance-cue effect appears to be strongest when peer effects are modest [13,18], possibly due to the increased difficulty of monitoring behavior in large groups. Additional studies can help explore the underlying mechanism through which surveillance cues operate. Our findings indicate that surveillance-cues effects should not readily be interpreted as evidence that altruistic behavior between strangers is maladaptive.

## Ethics statement

All experimental protocols were approved by the Internal Review Board at New York University Abu Dhabi (#014–2016) and by the Social Science Research Ethics Committee at United Arab Emirates University (ERS-2015-3212). Informed consent was obtained by participants in a way which would not make participants aware that their behavior in the field experiment was studied (see section A in S1 Appendix). Neither the existence nor the purpose of the field experiment that took place during the POSCE was revealed to participants at the end of the experiment. All aspects of our study, including the methods of consent and disclosure were carried out in accordance with relevant guidelines and regulations at United Arab Emirates University, and the IRB-approved protocols.

## Supporting information

S1 Appendix Details about experimental procedures, power calculations, robustness checks and surveys. (DOCX)

S1 Data (XLSX)
