# Supplementary material for: Surveillance cues do not enhance altruistic behavior among anonymous strangers in the field
Source: PLoS One. 2018 Aug 16;13(8):e0197959. doi: 10.1371/journal.pone.0197959 (PMC6095487; doi:10.1371/journal.pone.0197959)
Supplement: S1 Appendix — (DOCX) [file pone.0197959.s001.docx]

Supplementary Information for

**Surveillance cues do not enhance altruistic behavior**

**among anonymous strangers in the field**

Erik J. Koornneef, Aurelie Dariel, Iffat Elbarazi,

Ahmed R. Alsuwaidi, Paul B.M. Robben and Nikos Nikiforakis

This file includes the following sections:

A. Additional information on the experimental procedures and sample

B. Notes on the research assistants and robustness checks

C. Power calculations and sample size

D. The briefing note posted outside the room for the practice OSCE

E. The Observation Tool used by the RAs to evaluate the quality of hand hygiene

F. A picture of an ‘OSCE room’ used in the experiment

G. The pictures used in the experimental treatments

H. The WHO poster

I. Details of survey on hand hygiene

J. Survey and responses

K. Distribution of actions across treatments

L. Additional references

**A. Additional information on the experimental procedures and sample**

The experiment was conducted in a large university ranking amongst the best in the Gulf Corporation Council region – the United Arab Emirates University – over two consecutive days in April 2016. The local population is of interest as for millennia locals interacted in small tribal groups with their personal reputation constantly at stake. This situation changed only in the last 20-30 years, when the discovery of vast oil reserves led to the development of large metropolises and a dramatically different lifestyle. If the evolutionary legacy hypothesis is correct, then we would expect the local population to be as sensitive to any surveillance cues as any other population studied previously.

The data collection occurred between 9 am and 3 PM on both days. A medical student who expressed interest in the POSCE was told to arrive at the venue at a specific time, such that interaction with other students was minimized. Once at the venue, students were asked to sign a consent form. The consent form informed individuals that, after the POSCE, they would be invited to respond to a survey on “Creating a better understanding of healthcare regulation: Exploring the experience, views and perceptions of postgraduate medical students in the United Arab Emirates”. While the consent form mentioned the POSCE, the focus throughout was placed on an anonymous survey that would be conducted afterwards on views on healthcare regulation. The POSCE was presented as an opportunity to build individual experience prior to the survey such that students were not concerned that they would be studied. After signing the form, the student was randomly assigned into one of four *private* examination rooms. Each practice slot and room was randomly assigned into one of three treatments. A total of 114 students of a population of 330 eligible students participated in the POSCE (response rate 34.2%). 90 participants were female (79%) and 24 were male (21%). This is similar to the gender composition of the student body at the medical program where 73.6% of all graduate students is female and 26.4% is male.

To minimize reputational concerns, the patient-examination rooms for the POSCE were located in an isolated wing of the university were students typically do not have access to. Each participating student was told to arrive on site at a specific time such that interaction with other students outside the examination rooms prior to the POSCE was minimized. Upon arrival, each student was randomly assigned into one of four *private* examination rooms. Each practice slot and room was in turn randomly assigned into one of three treatments.

**B. Notes on the research assistants and robustness checks**

The simulated patients were eight research assistants (RAs). The RAs were always of the same gender as the medical student, and were randomly reassigned to examination rooms over the course of the day.

All RA’s completed a short training program, consisting of two online parts as well as a two-hour in person training. As part of the training program all participants successfully completed the online course from Hand Hygiene Australia ([http://www.hha.org.au/ LearningPackage/olp-home.aspx](http://www.hha.org.au/%20LearningPackage/olp-home.aspx)). The RA’s also completed a special course prepared by the authors. As part of this course, they were asked to use an ‘Observation Tool’ to observe and evaluate the hand-hygiene quality of a minimum of 10 pre-recorded cases. On average, the RA’s scored 95% of the observed behavior correctly. None of the RAs made contact with the participants before or after the experiment.

At any given point in the experiment, one RA waited for his/her blood pressure to be measured, and the other – seated at a faraway corner of the room – waited for his turn, while filling out a Sudoku book. In actuality, this individual was covertly monitoring the student’s HH practice. This implies that only one RA was responsible for recording a given observation. While multiple coders may have been desirable we decided against it as this would require adding more RAs in the room which could make students more self-aware. Importantly, however, the existence of one coder per observation should not bias our findings as there were several RAs overall, and each recorded roughly the same number of observations for all treatments at different points in time. Therefore, any systematic biases on the RA level (e.g., one being stricter in evaluating than another) should have no effect on our treatment estimates.

With one exception, all research assistants were unknown to the students and instructed not to share any personal information with participants. Due to being short of one RA, at the last minute, we recruited as an RA a staff member who may have been familiar to some of the participants. However, as one would expect given that RAs were randomly assigned to treatments, our results are unaffected if we exclude observations from this RA. In particular, with regards to the time spent washing, when using observations from all RAs, the average time spent washing was 21.5 seconds, and we find no statistically significant differences across treatments (Eyes vs. Baseline: *P*=0.69, *N*=71; Camera vs. Baseline: *P*=0.64, *N*=79; Mann-Whitney Test, two-tailed). Excluding the one RA who may have been known to some participants, the average time spent washing was 21 seconds, and again we find no statistically significant differences across treatments (Eyes vs. Baseline: *P*=0.78, *N*=60; Camera vs. Baseline: *P*=0.90, *N*=64; Mann-Whitney Test, two-tailed). With regards to the quality of hand coverage, when using observations from all RAs, there were no significant differences across treatments (Eyes vs. Baseline: *P*=0.99, *N*=71; Camera vs. Baseline: *P*=0.18, *N*=79; Mann-Whitney Test, two-tailed). Excluding the one RA who may have been known to some participants, we again find no significant treatment differences (Eyes vs. Baseline: *P*=0.76, *N*=60; Camera vs. Baseline: *P*=0.66, *N*=64; Mann-Whitney Test, two-tailed). Finally, with regards to the extent to which participants across treatments turned off the tap after washing their hands using a paper towel, we find no treatment differences whether we use observations from all RAs, (Eyes vs. Baseline: *P*=0.39, *N*=71; Camera vs. Baseline: *P*=0.90, *N*=79; Mann-Whitney Test, two-tailed) or if we exclude observations from that one RA (Eyes vs. Baseline: *P*=0.12, *N*=60; Camera vs. Baseline: *P*=0.77, *N*=64; Mann-Whitney Test, two-tailed).

The main role of the RAs for our research purposes was to accurately record the hand hygiene quality of the medical students prior to treating the patient. Once the student was in front of the washbasin, the RAs recorded the duration of the hand washing as well as the level of compliance with the recommended technique, using the Observation Tool. Although RAs were instructed to appear as bored and indifferent as possible, they were asked to gently prompt participants to wash their hands prior to taking their blood pressure in case the participants didn’t first wash their hands. RAs were instructed to do so in the most casual manner (e.g., by saying “Would you mind washing your hands first?”). This was essential for participants to be exposed to the experimental treatment, and occurred in 54 of the 114 instances (47%). As this could in principle activate participants’ reciprocity-based psychology, we repeat here the analysis using only observations from unprompted participants.

The likelihood a participant had to be prompted, as one might have expected, is unrelated to the treatment manipulation (*P=*0.77*, χ^2^*=0.68). Accordingly, our results are unaffected if we only use observations in which participants washed their hands without being prompted. In particular, we find no statistically significant differences across treatments when comparing the time spent washing (Eyes vs. Baseline: *P*=0.91, *N*=36; Camera vs. Baseline: *P*=0.49, *N*=36; Mann-Whitney Test, two-tailed), the quality of hand coverage (Eyes vs. Baseline: *P*=0.64, *N*=36; Camera vs. Baseline: *P*=1.00, *N*=36; Mann-Whitney Test, two-tailed), and the extent to which participants across treatments turned off the tap after washing their hands using a paper towel (Eyes vs. Baseline: *P*=0.14, *N*=36; Camera vs. Baseline: *P*=0.88, *N*=36; Mann-Whitney Test, two-tailed).

**C. Power calculations and sample size**

Convention prescribes that the sample size is such that a given treatment effect will be significant at the 5% level, 80% of the time (that is, the power of the test is 80%). Our study is the first to explore the impact of surveillance cues on the quality of hand hygiene in a natural field setting. We therefore have no prior on the potential size of the treatment effect (if any). To ensure that our statistical tests would be sufficiently powered we used the following approach to calculate our sample size. As a benchmark for the average quality of hand hygiene in our baseline condition we used the average compliance rate across a number of observational studies, which is 40% (Boyce and Pittet, 2002). As mentioned, the photograph of the male eyes in our experiment was taken from (23) who found an effect of 122%. Given these numbers, we need a sample of 15 participants per treatment to detect a significant difference at the 5% level, 80% of the time. Given our sample, we have a power of 99.72%.

**D.** **Briefing note posted outside room**

**Practice OSCE**

***Blood Pressure Measurement***

**AIM**

The aim of this station is to assess your ability to measure blood pressure correctly whilst communicating appropriately with the patient.

**TASK**

Measure the patient's blood pressure. There is no need to take a history from the patient, but you may ask questions and give any explanations and advice that you feel are appropriate.

**SCENARIO**

You do not know this patient and there is nothing of significance in his/her records.

**PATIENT**

There will be two simulated patients in the room. They will take it in turns to be the “patient”.

**EQUIPMENT**

- An aneroid sphygmomanometer and a mercury sphygmomanometer.
- A standard cuff (size 23cm x 12 cm)
- A stethoscope

**WHEN YOU HAVE FINISHED**

You may tell the patient what the blood pressure is.

**E. The Observation Tool used by the RAs to evaluate the quality of hand hygiene**

| **Clinical Skills Observation Tool - Blood Pressure Measurement** | | | | |
| --- | --- | --- | --- | --- |
| Participant ID: | |  | | |
| Research Assistant ID: | |  | | |
| **Part 1: Preparation** | | **Performed adequately & completely** | **Attempted but inadequate / incomplete** | **Not attempted** |
| 1 | Introduces self (no identifying information must be given) |  |  |  |
| 2 | Checks that the patient has been resting for 3 minutes and has not eaten for 30 minutes, if yes, ask patient for their consent to carry out the procedure |  |  |  |
| **Part 2: Hand hygiene** | | **Performed adequately & completely** | **Attempted but inadequate / incomplete** | **Not attempted** |
| 3 | Participant needed a prompt/reminder to wash hands. | YES / NO | | |
| 4 | Coverage – are all hand surfaces cleaned adequately (i.e. did the participant adequately follow all the steps) |  |  |  |
| 5 | Dries hands with paper towel. |  |  |  |
| 6 | Turns off tap with paper towel |  |  |  |
| 7 | Throws paper towel in correct bin | YES / NO | | |
| 8 | **Total time spent washing hands** (in seconds, round up to the nearest 5 sec.) Note: start clock when tap is turned on and stop clock when tap is turned off |  | | |
| **Part 3: Blood pressure measurement** | | **Performed adequately & completely** | **Attempted but inadequate / incomplete** | **Not attempted** |
| 9 | Assists the patient into a comfortable position. Ensure that the arm is at heart level and resting on a suitable firm surface |  |  |  |
| **Part 4: Hand hygiene** | | **Performed adequately & completely** | **Attempted but inadequate / incomplete** | **Not attempted** |
| 10 | Participant needed a prompt/reminder to wash hands. | YES / NO | | |
| 11 | Coverage – are all hand surfaces cleaned adequately (i.e. did the participant adequately follow all the steps) |  |  |  |
| 12 | Dries hands with paper towel. |  |  |  |
| 13 | Turns off tap with paper towel |  |  |  |
| 14 | Throws paper towel in correct bin | YES / NO | | |
| 15 | **Total time spent washing hands** (in seconds, round up to the nearest 5 sec.) Note: start clock when tap is turned on and stop clock when tap is turned off |  | | |
| **Additional Comments/Observations:** | | | | |
| Please circle one number to indicate your level of agreement with the following statement regarding the candidate’s performance:“The candidate demonstrated a professional approach that included concern for the patient and maintained patient dignity” **Strongly agree Strongly disagree**   1. 4 3 2 1 | | | | |

**F. A picture of an ‘OSCE room’ used in the experiment**


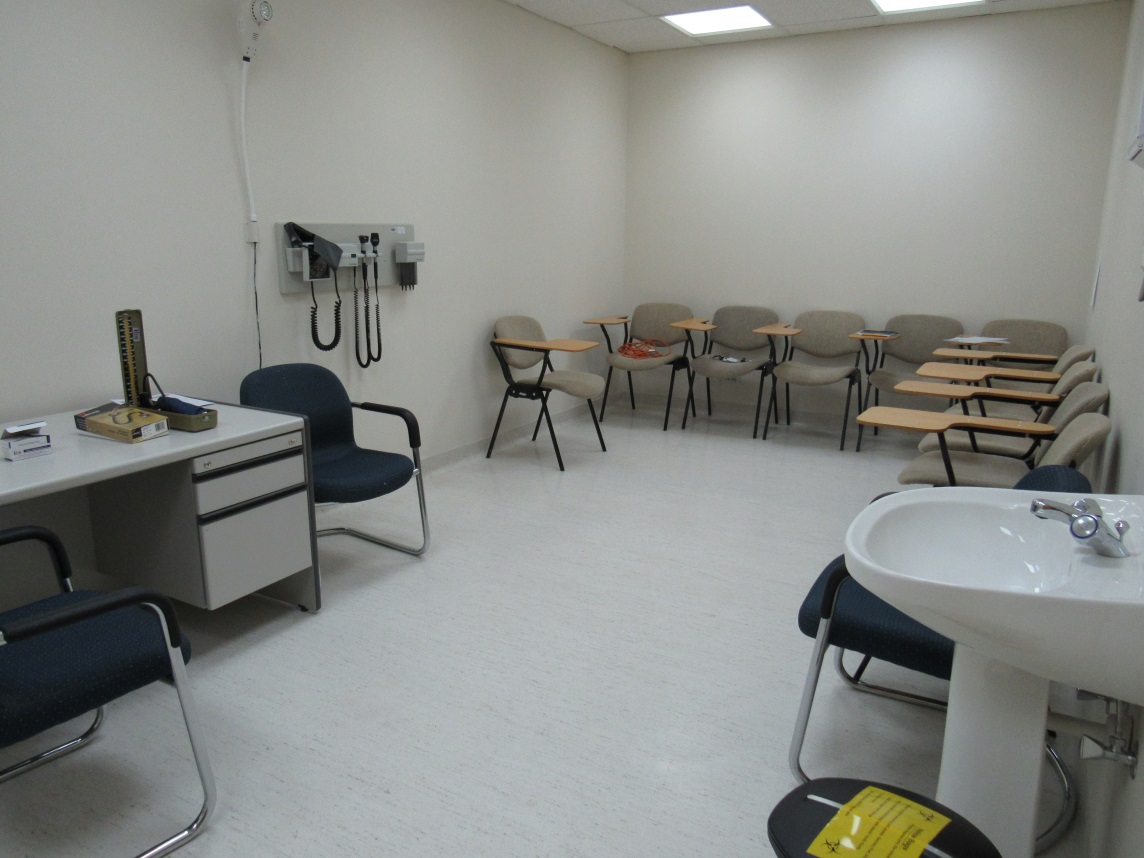


**G. The pictures used in the experimental treatments**

| Baseline | Eyes | Camera |
| --- | --- | --- |
| 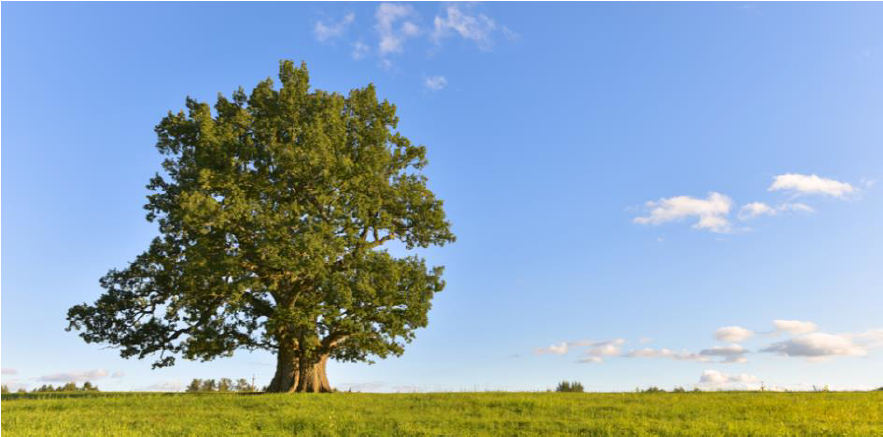 | 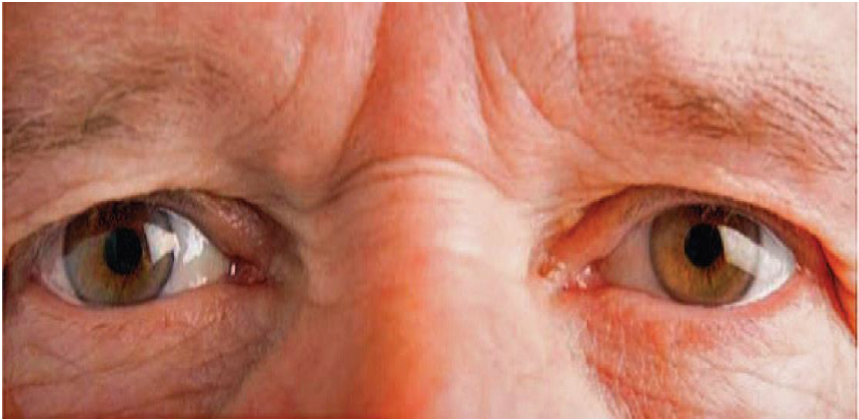 | 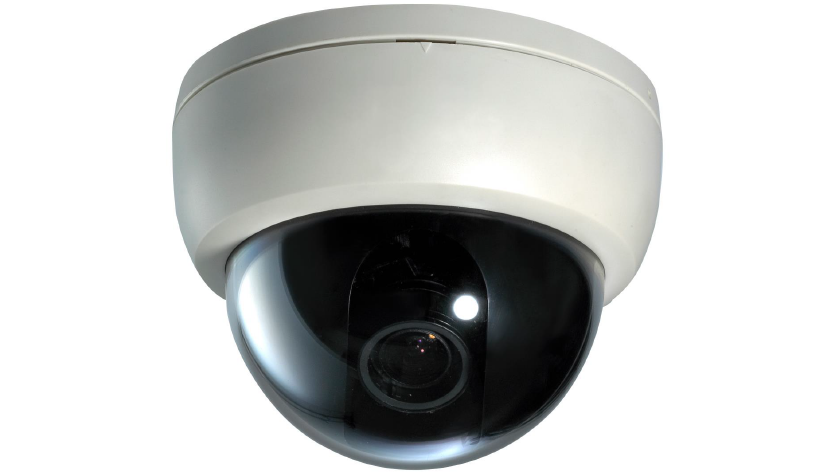 |

**H. The WHO poster**


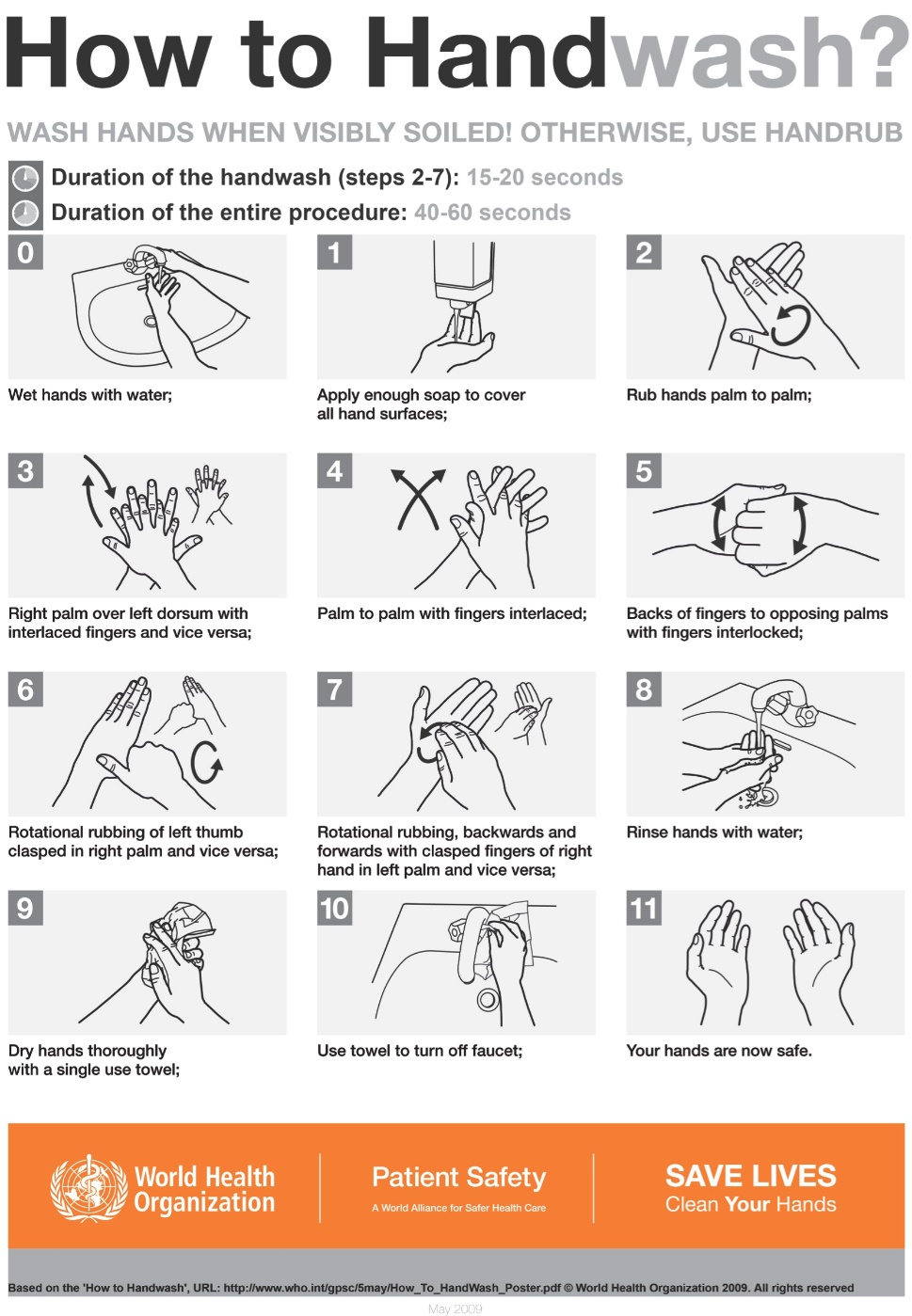


**I. Details of the survey on hand hygiene**

To investigate whether hand hygiene prior to treating a patient in our experiment is an altruistic act we administered a survey to 100 medical students with the same level of training as those who participated in our experiment. Respondents were presented with a vignette designed to mimic the situation and incentives in our experiment, and asked whether they would wash their hands prior to treating the patient or not, and the reasons for their decisions. The survey also included a question to evaluate our claim that the quality of hand-hygiene matters when it comes to reducing infection by asking participants whether they agree that washing hands for longer reduces the risk of infection for the patient. The full survey is available in S10.

The survey was administered in the last week of February 2018. Respondents were students in preclinical years, from years 3-5 of the MD Program. They were approached either in their classes (7 classes) or in the clinical lab skills sessions (4 sessions) and were invited to fill in the questionnaire. Year 5 students were approached in their clerkship rotation. Those who agreed to participate were provided with the survey and instructed to drop the filled-out survey at a closed box outside the office of the education coordinator. Most students agreed to participate. The few who chose not to participate had no information about the content of the survey.

The survey presented students with a simple vignette designed to mimic the situation and incentives in our experiment. To avoid confusing respondents who were still unfamiliar with the OSCE, we refrained from referring to either an OSCE or a POSCE in the vignette, placing instead the interaction in a medical environment that would be familiar to all medical students. Respondents were then asked whether they would wash their hands prior to treating the patient or not, and the reasons for their decisions. The different responses appeared in a random order.

The responses indicate clearly that the main concern driving hand hygiene prior to treating a patient appears to be a desire to do no harm to him/her. We can reject the hypothesis that respondents neither agree nor disagree with the statement that they would wash their hands to avoid harming the patient in favor of the alternative hypothesis that they agree with this reason (Wilcoxon, *p*<0.01, *N*=87). Participants are significantly more likely to agree with this reason than with any of the other reasons (Wilcoxon, *p*<0.01, *N*=87, for all pairwise comparisons). Only 12.4% of respondents who said they would wash their hands before taking the patient’s blood pressure had a reason with which they agreed more strongly than avoiding harm.

The survey also included a question to evaluate our claim that washing hands for longer is believed to reduce the risk of infection. We find clear evidence that respondents believe the duration of HH is an important determinant of the likelihood of infection. We can reject the hypothesis that respondents neither agree nor disagree with the statement that “A doctor who washes her hands for 30 seconds is less likely to infect a patient than a doctor who washes her hands for 10 seconds” in favor of the alternative that they agree (Wilcoxon, *p*<0.01, *N*=87).

**J. Survey and responses**

**Welcome**

We are a team of researchers from United Arab Emirates University, New York University in Abu Dhabi and Erasmus University in Rotterdam. We are conducting a short survey regarding hand hygiene among practitioners. Please spare us a few minutes of your time to answer 3 short questions.

**Consider the following situation**

You have a 10-minute appointment with a patient in your office. This patient is not one of your regular patients. The patient normally sees another doctor, but today this doctor is unavailable. There is nothing of significance in the patient’s record. Since this is the first time you see the patient you decide to take her/his blood pressure. You have just arrived at your practice for the appointment after driving thirty minutes from your home.

**Question 1 (*N*=100)**

Would you wash your hands **before** taking the patient's blood pressure? (tick one answer)

Yes 87%

No 13%

If you answered “Yes”, please go to page 2, otherwise, please go straight to page 3.

**Question 2 [Only for those who answered YES]: (*N*=87)**

Please state how much you agree with the following reasons for washing your hands **before** taking the patient's blood pressure by ticking one of the answers.

*[Note: entries are the percentage of respondents who gave a particular answer, except in the last column which presents the mean response. *** and ** indicate that a Wilcoxon test rejects the null hypothesis that the mean is not different from 0 at the 1% and 5% level, respectively]*

|  | Strongly disagree  (-2) | Somewhat disagree  (-1) | Neither agree nor disagree (0) | Somewhat agree  (1) | Strongly agree  (2) | Mean  response |
| --- | --- | --- | --- | --- | --- | --- |
| 1. I wash my hands because I wish to do no harm to the patient by infecting them | 0.00% | 1.15% | 2.30% | 12.64% | 83.91% | 1.79*** |
| 2. I wash my hands because that is what all other clinical practitioners do | 13.95% | 15.12% | 39.53% | 26.74% | 4.65% | -0.07 |
| 3. I wash my hands because it reduces the likelihood the patient complains about me | 25.58% | 15.12% | 29.07% | 24.42% | 5.81% | -0.30** |
| 4. I wash my hands because that is what all clinical practitioners are expected to do | 5.81% | 3.49% | 19.77% | 36.05% | 34.88% | 0.91*** |
| 5. I wash my hands because I want to be a good ambassador for my practice/hospital | 2.33% | 2.33% | 9.30% | 24.42% | 61.63% | 1.41*** |

Please state any other reasons for washing your hands. ________________

(Note: 26.4% of respondents provided responses here, however, the vast majority of them simply provided variants of the “do no harm” response, suggesting they used the opportunity to elaborate on their reasons for washing their hands.)

**Question 2 [Only for those who answered NO]: (*N*=13)**

Please state how much you agree with the following reasons for **not** washing your hands **before** taking the patient's blood pressure by ticking one of the answers.

*[Note: entries are the percentage of respondents who gave a particular answer, except in the last column which presents the mean response. *** and ** indicate that a Wilcoxon test rejects the null hypothesis that the mean is not different from 0 at the 1% and 5% level, respectively]*

|  | Strongly disagree  (-2) | Somewhat disagree  (-1) | Neither agree nor disagree (0) | Somewhat agree  (1) | Strongly agree  (2) | Mean  response |
| --- | --- | --- | --- | --- | --- | --- |
| 1. Not washing my hands will not harm the patient | 23.08% | 15.38% | 30.77% | 15.38% | 15.38% | -0.15 |
| 2. Not washing my hands will reduce the consultation time | 53.85% | 15.38% | 7.69% | 23.08% | 0.00% | -1** |
| 3. Not washing my hands speeds up the process of building immunity to disease for me | 30.77% | 15.38% | 46.15% | 0.00% | 7.69% | -0.62* |
| 4. Washing my hands irritates my skin/ | 53.85% | 15.38% | 15.38% | 15.38% | 0.00% | -1.08** |
|  |  |  |  |  |  |  |

Please state any other reasons for not washing your hands. ______

(Note: 76.9% of respondents provided responses here. The majority of them said that hand washing was not necessary and hand sanitization was sufficient. Among the others, some mentioned that hand washing was not necessary, time pressure or that they would forget.)

**Question 3 [for all respondents]: (*N*=93)**

Please state how much you agree with the following statement by ticking one of the answers:

*“A doctor who washes her hands for 30 seconds is less likely to infect a patient than a doctor who washes her hands for 10 seconds.”*

| Strongly disagree  3.23% | Somewhat disagree  7.53% | Neither agree nor disagree  10.75% | Somewhat agree  39.78% | Strongly agree  37.63 | Mean  1.01*** |
| --- | --- | --- | --- | --- | --- |
|  |  |  |  |  |  |

*[Note: entries are the percentage of respondents who gave a particular answer, except in the last column which presents the mean response. *** and ** indicate that a Wilcoxon test rejects the null hypothesis that the mean is not different from 0 at the 1% and 5% level, respectively]*

**Thank you!**

**K. Distribution of actions across treatments**

**L. Additional references**

Boyce J.M., & Pittet, D. (2002). Guideline for Hand Hygiene in Health-Care Settings. *Morbidity and Mortality Weekly Report,* 51, 1-45. Available at: [https://www.cdc.gov/ mmwr/PDF/rr/rr5116.pdf](https://www.cdc.gov/%20mmwr/PDF/rr/rr5116.pdf)
